# The bond and retention of Chinese seafarers for international shipping companies: a survey report

**DOI:** 10.1186/s41072-021-00087-1

**Published:** 2021-04-20

**Authors:** Bin Wu, Glory Gu, Chris James Carter

**Affiliations:** 1 Haydn Green Institute for Innovation and Entrepreneurship, University of Nottingham, Nottingham, UK; 2 FLEET Management Limited, Hong Kong, Hong Kong

**Keywords:** Bond of global seafarers, International shipping companies, Seafaring career, Retention of seafarers, Chinese exported seafarers

## Abstract

The shortage of junior seafarers in China in recent years raises a salient question as to how international shipping companies can improve retention rates among Chinese crews. This issue has become increasingly prominent in the context of a global lockdown resulting from the Covid-19 pandemic. This paper examines the dilemma through the lens of the “bond” between seafarers and the shipping companies they service, a term used to reflect the need to recognise, consent and integrate into management systems, safety culture, and organizational values. The value of this bond concept is investigated in a survey of Chinese crews (*N* = 318). The paper aims to reveal the features and underlying factors of the bond, and its influence on needs, perceptions and seafaring careers in foreign shipping companies. The study finds that the majority of respondents do not have a bond with their shipping company, but typically do wish to develop one. Furthermore, this form of attachment appears to be closely related to career satisfaction and retention. To address the shortage of junior seafarers in China, we call for the development of mutual trust, respect and shared values between global seafarers and international shipping companies. A number of policy recommendations are provided.

## Introduction

As the largest seafarer supply country in the world (BIMCO/ICS 2016), China is facing a severe challenge in securing a sufficient supply of junior officers for international shipping. According to the latest *China Seafarer Development Report 2019,* the number of exported seafarers who service foreign shipping companies was 137,569 in 2019; an increase of 6.5% annually, or 16.6% compared with 2015 figures (MoT 2020). The supply of 3rd officers and 3rd engineers, however, has decreased by 26.5 and 25.4%, respectively, in 2019 though the enrolment of new students in Maritime Education and Training (MET) institutes nationwide has increased by 26.1% from 14,960 in 2015 to 18,864 in 2019 (MoT 2020). An important factor appears to be declining interest amongst this cohort of MET graduates in working onboard ships. According to statistics from the top ten MET universities and colleges in China, the average embarkation rate of graduates has declined from 36.5% in 2017 to 28.0% in 2019 and from 31% to 26.3% within the top four MET universities during the same period (MoT 2020).

Furthermore, the shortage of junior seafarer supply has been exacerbated by the Covid-19 pandemic, which has blocked the normal exchange of crews and had a significant impact on both Chinese seafarers and their families. As a result, there has been a marked shortfall in the supply of Chinese seafarers, leading to rapid growth of Chinese seafarer salaries since February 2020.^1^

Shortage of quality seafarer officers is not an entirely novel issue in international shipping, having been precipitated by a number of factors in the twenty-first Century, including the expansion of international merchant  fleets, high mobility and several disadvantages of the seafaring occupation compared with professional shore-based jobs (e.g. social isolation, needing to leave families behind). The shortage of Chinese junior officers in recent years, however, may reflect some fundamental issues in the Chinese seafarer exporting system, for which there are a number of explanations. Given that more than eight million students graduate from Chinese universities or colleges each year, a crucial factor responsible for the shortage of Chinese seafarer supply relates to the lack of public awareness, appropriate understanding, faviourable policy and media environment for the development of seafaring careers. Other factors include a decline in the wage gap between shore-based jobs and seafarers, leading to diminished attractiveness of exported seafarers^2^; deficiency of English competency among Chinese graduates (Fan et al. 2018; Tang et al. 2016**)**, and institutional barriers that do not allow foreign shipping companies to directly recruit Chinese seafarers (Wu 2004; Tang et al. 2016; Tang and Zhang 2019; Wu et al. 2006, 2007; Zhao et al. 2020**)**.

However, we argue that such explanations ignore an important fact that shipping companies are not homogeneous in how they treat Chinese crews, leading to differences in retention rates and levels of appreciation from Chinese seafarers. To fill this knowledge gap, this paper draws attention to the phenomenon of the bond between Chinese-exported seafarers and international shipping companies to reveal their recognition, psychological status and intention to follow a seafaring career and provide long-term service to trusted shipping companies. In particular, this paper aims to address the following questions: 1) What are the features and underlying factors of the bond between Chinese-exported seafarers and international shipping companies? 2) How does the bond influence their needs, perceptions and seafaring careers in the international shipping? 3) What are the policy implications for international shipping companies to help attract and retain committed and highly skilled seafarers for long-term service?

The arguments are organised across six sections. Section 2 provides a brief background and methodology of survey design and delivery. Section 3 provides the definition and measurement of “bond” and associated profiles of the respondents to the survey. Section 4 focuses on the distribution of the bond among respondents and key factors underpinning it, and Section 5 illustrates features of different groups in terms of needs, perceptions and career planning. The paper closes with a summary of research findings and policy implementations.

## Background and methodology of the research design

The inspiration for this paper can be traced back to 2006, when the authors attended the first Shengzhen International Maritime Forum, organised under the theme: “How to develop quality seafarers to meet the increasing demand in the global labour markets” (Wu 2006). In the subsequent Winter of 2006–07, the first author of this paper spent 73 days onboard two international merchant vessels with Chinese crews (a Bulk Carrier with Singapore Flag, a Chemical Tanker with Norwegian Flag) to learn about their attitudes, stories, and opinions on seafaring careers, as well as their perceptions and comments on their relationship with the shipping company (Sampson and Wu 2007). For updated information and the latest development of Chinese seafarers in foreign shipping companies, a second field research study was undertaken in China between 2017 and 2018 using a combination of quantitative and qualitative methods.

The qualitative research took place in Beijing, Shanghai and Dalian in the summer of 2017 for two weeks. It involved a series of visits to two crew agencies (one state-owned and one private) and three representatives of shipping company offices (Japanese, Hong Kong, and other Asian locations respectively) to: 1) observe and communicate with official Chinese crews who were preparing their voyage on board ships; 2) conduct interviews with staff and Chinese crews to learn the processes and challenging issues related to the theme of this project; 3) develop and test hypotheses related to career development of Chinese seafarers onboard foreign ships; 4) explore the access to and pathways for the dissemination of surveys to Chinese crews as a later phase of the research. With a broad theme of career development and constraints upon Chinese seafarers, representatives from foreign shipping companies, crewing agencies and Chinese seafarers were asked to offer their opinions, comments and explanations in one-hour unstructured interviews. In total, 12 interviews were conducted, of which three were with active seafarers (one cadet and two senior officers). The remaining eight interviews involved shipping company representatives and crewing agency managers, of which one was a non-Chinese Captain working in a Chinese crewing agency responsible for training in English language and competence assessment.

The transcription and analysis of the 12 interviews led to the emergence of a common theme of interest and the subsequent focus of this report: the bond between Chinese seafarers and foreign shipping companies, and a draft survey to examine this. The survey consisted of four sections: First, personal background and seafaring mobility experience; second, satisfaction and plans for a seafaring career; third, perceptions and evaluation of the exported seafarer experience; and fourth, comments on the trends and performance of Chinese exported seafarers. The survey was primarily focused upon capturing the view of respondents with regards to their bond with shipping companies, its relationship with their movitation as an exported seafarer and impact on satisfaction and career development plans. The voluntary and anonymous nature of participation was emphasised at the outset of the survey, and no identifiable information relating to participating shipping companies or crew agencies was revealed.

A pilot survey was conducted in a foreign shipping company involving 80 Chinese crews. Based upon the preliminary analysis and assessment on the results from the pilot survey, a further amendment was made to examine the mobility experience of the seafarers and elaborate on factors related to their selection of foreign companies. After this amendment, the finalised survey was disseminated through crewing agencies across China.

To minimise sampling bias, the following criteria were applied in disseminating and selecting valid surveys: 1) Whenever possible, comprehensive coverage of international fleets from Western, Chinese (e.g. Hong Kong, Singapore and Taiwan) and other Asian locations (e.g. Japan, South Korea and India) was attempted; 2) The sample included different types of Chinese crewing agencies in terms of ownership (state vs. private), geographic location (north and south China), and size (large: > 4000, medium: 1000–4000, small: < 1000 in terms of Chinese crews); 3) The sample included all types of Chinese crews in relation to employment status (i.e. state-owned enterprise (SOE) employees, agent seafarers with long contracts, and “free seamen”) and rank (senior, junior officers and ratings, referring to unskilled/semi-skilled crews working in the deck or engine room); 4) The sample included active seafarers onboard or on-leave; 5) The sample included no more than 20 respondents for each fleet or medium crewing agencies.

Adhering to the above criteria, the finalised surveys were disseminated between August and October 2018 via a range of channels, including shipping companies, crewing agencies and training courses attended by officers on leave. After a process of validity checking against the quality of the survey and taking into account the principles above, a total of 318 surveys were included in the dataset for further analysis and presentation in this report. However, we do not claim that the sample included in our survey can represent the full distribution of Chinese exporting seafarers, and acknowledge elements of sample bias, including: a relative overemphasis in the sample of seafarers from coastal regions, and less attention paid to SOE companies: an important source of Chinese exporting seafarers.

## Definition of bond and profiles of respondents

The term *bond* in this paper is used to refer to the psychological phenomenon amongst Chinese-exported seafarers, who want to share their personal identity with, or are willing to become, a member of foreign shipping companies that they are serving, despite there being no direct, formal employment contract between them. Under the current regulation system in China, no labour market is open to foreign employers directly, so they have to go through the channel of intermediary labour brokers to search, recruit and sign both employment and boarding (voyage) contracts with foreign companies. In the case of exported seafarers, labour brokers are those licensing to Chinese crew agencies (other sectors involve Chinese labour exporting include, among others, nurses and air crews).

Despite the complicated and tripartite relationship described above, bonding was a common topic of interest mentioned by shipping company representatives, Chinese crewing agents and seafarers. The following is a quote reflecting the concept of bonding among Chinese crews:

“To be honest, I don’t think that my belonging to a foreign shipping company is strong at moment although I desire to be recognized by a company as its employee for a long-term service.

[*Could you say that you belong to this manning agent*?]

Not sure because it provides the expatriate services only.

[*could you say that you belong to your current ship company*?],

Of course not. It is not good at all for a seafarer who doesn’t have a feeling of belonging to. I wish to become an employee of a foreign shipping company eventually not just in name but also in welfare and pay package” (Mr. Wang, First Officer)

The desire to be a member of a shipping company is not merely for the purpose of a long-term service, but involves shared values with a shipping company as reflected by the quote below:

“In fact, I was treated well in the previous company in terms of pay and promotion, but I didn't have the feeling of belonging to it. This is because the way the company was run was totally based upon commercial principles. As a senior officer, however, I am concerned about the safety of the ship and crews, and also have a long-term perspective of our seafaring career in the company. In contrast, the current ship company with a long history has a good reputation in international shipping. What you can see is honesty, seriousness and high standards, which makes me feel just like a member of the company. This was reason why I decided to move from that company to the current one”. (Chief Engineer Cheng)

The above quote suggests that shipping companies may have different approaches to the bond of Chinese seafarers. This is expressed by the two contrasting opinions:

“We want to keep seafarers to continue to work with us so that we keep their ‘heart’ (commitment and loyalty) with us. If their ‘heart’ is not here, never put them onboard, not only because it involves the safety of hundreds of millions of US Dollars of assets, but also the safety of over twenty lives and their families.” (Capt. L, a Japanese shipping company representative)

“In fact, foreign shipowners avoid talking about the issue of the ‘feeling of belonging’, because they are unwilling to give Chinese crews a promise of long-term employment. For this reason, the shipowner’s strategy is to transfer the risk to Chinese crew agencies”. (Capt. W, a private crew agency manager)

Nonetheless, it is possible to identify a trend with shipping companies paying increasing attention to the bond of Chinese seafarers, which the quote from a shipping company representative exemplifies:

“There is a close correlation between appreciation and accountability among Chinese crews. There are two types of responsibility: one comes from the shipping company and crew members have no choice but follow (required behaviour), another comes from seafarer’s initiative (voluntary behaviour), who are willing to do something for the company. In recent years, there is an increase of people holding the second attitudes. Our company has begun to pay attention to develop their belonging and appreciation among Chinese crews, in particular these key persons [i.e. Top 4: Captain, Chief Engineer, First Officer, Second Engineer]”.

Two conclusions can be drawn from the above quotes. First, bonding is of common interest and is now regularly debated among shipping companies, with Chinese agents and crews showing that different people may have different approaches. Second, bonding offers an effective means for us to recognise and distinguish exporting seafarers into different groups according to their attitudes, perceptions and psychological attributes in relation to the relationship with foreign shipping companies.

To measure this bond, we provided five options to the survey question, “Do you feel that you are an actual member of a shipping company, despite having no formal employment contract signed with it?”: 1) Yes, I feel, 2) Yes, I wish, 3) I am not sure, 4) I don’t think so, 5) Difficult to say.

Turning to the profiles of respondents, the mean age of respondents in the sample was 35.3 years old, of which 78.5% were from the coastal region, 17.3% from the central region and only 4.2% from the western region. At the time of the survey, 74.6% of respondents were married, and 70.5% of their parents are rural hukou holders, leaving the rest as urban hukou. Furthermore, 19% held a university degree, 47.6% had vocational education, leaving one-third (33.3%) who completed middle school education (mainly senior high school). In term of rank distribution, senior and junior officers were similar in size, sharing 62%, leaving the rest (38%) as ratings. The average length of seafaring career and their servicing as exported seafarers were 10.9 years and 7.5 years, respectively. Table 1 provides a summary of the sample of the seafarers’ profiles:

**Table 1:** Profiles of respondents by age, education, rank, seafaring and exporting length (years)

*Age*	*%*	*Education*	*%*	*Rank*	*%*	*Seafaring*	*%*	*Exporting*	*%*
< 30	30.8	University	19.0	Senior	31.8	<=5	31.0	<=5	46.3
30–39	30.2	Vocation	47.6	Junior	30.2	6–10	28.2	6–10	31.5
> = 40	38.1	M School	33.3	Ratings	38.1	> 10	40.8	> 10	22.2

With regards to employment status (defined as a long-term contract signed with SOE shipping company or crewing agency), Table 2 shows that 44.4% define themselves as “free seaman”, the largest group among respondents, followed by those from crew agencies (38.9%) and SOE companies (16.7%). The average age of respondents from crew agencies was 31.8 years old; the youngest group compared with 38.3 years old of free seamen, intermediated by seafarers from SOE companies at 35.8 years old. With regards to the length of current employment status, on average, those from SOE companies reported 11.1 years, those from crewing agencies 6.8 years, while free seamen reported 8.8 years. It is noted that free seamen represent 52.5% of senior officers and ratings, while seafarers depending on crewing agencies represent nearly 60% of the junior officers.

**Table 2:** Seafarers’ age, duration and distribution by employment status and rank (years, %)

*Category*	*No.*	*%*	*Age*	*Duration*	*Senior*	*Junior*	*Ratings*
SOE company	52	16.7	35.8	11.1	21.2	15.2	14.2
Crew agency	121	38.9	31.8	6.8	26.3	**59.8**	33.3
Free seamen	138	44.4	38.3	8.8	**52.5**	25.0	**52.5**
Total/Mean	311	100	35.4	8.4	100	100	100

*Note*: bold is to highlight

For mobility experience, the respondents can be distinguished from two interwoven respects: movement between foreign ship companies, and movement between crew agencies. Table 3 shows 48.2% of the respondents had kept their job in their current ship company (“one only” category), which was slightly higher than those in the same category in the current crewing agency (53.4%). The mobility rate, however, varied significantly, depending upon the employment status and the rank. For instance, seafarers from SOE companies and crewing agencies are more likely to be sent by the same crew agency (around three-quarters) and also stay in the same foreign company vessels (over 50%). By contrast, only 38% of free seamen stay in one shipping company and more than 70% experienced moving between two or more crewing agencies. Furthermore, senior officers reported higher mobility, as only 30% of the former stay in the same shipping company, 25% lower than other ranks. It is noted that junior officers were more likely to stay in one crewing agency (over 70%) in order to develop their seafaring career.

**Table 3:** Mobility of respondents across ship company and crew agency (%)

*Category*	*Item*	*Ship company*			*Crew agent*		
		*One only*	*2 to 3*	*> 3*	*One only*	*2 to 3*	*> 3*
Employment	SOE company	**51.3**	25.6	23.1	**77.1**	14.3	8.6
	Crew agency	**57.4**	32.4	10.2	**74.6**	20.2	5.3
	Free seamen	38.0	36.1	**25.9**	28.2	**54.0**	17.7
Rank	Senior officer	30.1	33.7	**36.1**	37.9	43.7	**28.4**
	Junior officer	**55.1**	35.9	9.0	**71.1**	26.5	2.4
	Rating	**56.6**	30.3	13.1	52.3	35.8	11.9
Mean		48.2	32.9	18.8	53.4	35.5	11.1

## Distribution of bonded seafarers and key underpinning factors

Table 4 shows that 41.3% confirm that they feel a bond, while the remainder (58.7%) do not have such a feeling. Despite the majority of respondents reporting a lack of bond, 27.1% expressed their desire to possess such a bond with the shipping companies. As a result, we classify 68.4% of respondents as falling into the category of “***bonded”***, nearly one in five (19.7%) in ***neutral*** (including “not sure” and “difficult to say” responses) and the remainder (11.9%) in ***detached*** (“don’t think so”). The majority (two-thirds) of exported seafarers in our survey reported a bond with shipping companies, to some extent.

**Table 4:** Feelings of membership in relation to the shipping company

	*No*	*%*	Cum*. %*	*Category*
Yes, I feel	128	41.3	41.3	*Bonded*
Yes, I wish	84	27.1	68.4	
Not sure	45	14.5	82.9	*Neutral*
Don’t think so	37	11.9	94.8	*Detached*
Difficult to say	16	5.2	100	*Neutral*
Total	310	100	–	–

Table 5 presents the characteristics of the different groups in relation to the bond and the significant differences between respondents in terms of education, employment status, mobility and multicultural crewing experience, and cultural background of the shipping company.

**Table 5:** Distribution of responses by type of bonding and various factors

*Factor*	*Item*	*Detached*	*Neutral*	*Bonded*
Mobility: shipping company	One only	8.1	14.6	**77.2**
	2–3 times	17.4	23.3	59.3
	> 3 times	8.0	18.0	**74.0**
Mobility: crewing agent	One only	8.8	16.2	**75.0**
	2–3 times	14.3	27.6	58.2
	> 3 times	12.9	6.5	**80.6**
Employment status	SOE employee	13.7	23.5	62.7
	Agent seafarer	6.8	16.1	**77.1**
	Free seaman	16.4	20.9	62.7
Education	University	16.9	11.9	71.2
	Vocational	6.8	18.9	**74.3**
	M school	16.8	25.7	57.4
Multination experience	Yes	10.6	14.8	**74.6**
	No	12.5	26.8	60.7
Culture of ship company	Chinese	7.9	18.1	**74.0**
	Non-Chinese	17.3	21.8	60.9
Total		11.9	19.7	68.4

*Note*: bold is to highlight differences between bonded and other groups

A number of observations can be drawn from Table 5. First, bonding is related to the retention rate, which is indicated from the finding that 77.2% of the respondents without mobility experience (one shipping company only) fell into the category of the *bonded* group, nearly 20% higher than those who had mobility experience of 2 or 3 shipping companies. However, the reverse was not true, as nearly three-quarters of highly mobilised seafarers (> 3 times) reported considerable levels of attachment. Indeed, correlation analysis indicates that greater reported attachment was significantly related to lower mobility with shipping companies [(*r* = .13, *p* < .05)], though the relationship was not statistically significant for the mobility among crewing agents [(*r* = .11, *p* = .08).]

Second, bonding was also closely related to the partnership between shipping companies and crewing agencies with regards to the following observations:

1. Over three-quarters (77.1%) of agent seafarers fell into the *bonded* group, 15% higher than their counterparts from SOE seafarers and free seamen;
2. Three-quarters of respondents who reported a stable relationship with the crewing agency (i.e. one agency only) fell into the *bonded* groups, a percentage much higher than the average of 68.4%. Post-hoc analysis of variance indicated that free seamen reported significantly lower attachment compared to agent employees (*p* < .001) but not SOE employees (*p* = .33), with employment status a significant factor determining level of attachment [F(2, 300) = 5.83, *p* < .01].

Third, those with experience of high mobility among shipping companies and crewing agencies, did not necessary fall into the *neutral* or *detached* groups. Rather, such experience provides opportunities for them to compare differences between shipping companies or crewing agencies, and then “settle-down” and develop their bond with their current shipping company. The findings of our survey shown in Table 5 support this claim, as over three-quarters and over 80% of high mobile respondents fell into bonded group.

Fourth, in relation to factors underpinning bonding, Table 5 shows that:

1. The number of graduates from universities (71.2%) and vocational institutes (74.3%), in particular, reported greater bonding than those from secondary education (57.4%). Post-hoc analysis of variance indicated that respondents with school level education reported significantly weaker bonding compared to those with vocational qualifications (*p* < .001) but not those with university education (*p* = .08). Overall, education level is a significant factor in determining the level of bond [F(2, 305) = 5.64, *p* < .01)].
2. Multicultural working experience could also contribute to bonding as three-quarters of respondents were in the bonded group, nearly 15% higher than those without such experience;
3. Among shipping companies, respondents from Chinese cultural background companies were also 15% higher than their counterparts working for Western or other Asian shipping companies. Univariate analysis confirms that respondents with Chinese shipping experience reported significantly greater bonding to their employer compared to those without, F(1, 302) = 10.35, *p* < .001. In contrast, respondents with other Asian shipping experience reported significantly weaker bonding with their shipping companies compared to those without, F(1, 302) = 6.40, *p* < .01, while Western shipping experience was found to have a significant effect on bonding.
4. There was no significant correlation between bonding and age, rank, region of home, marriage status, length of seafaring and service as exported seafarers.

## Needs, perceptions and planning of seafaring career

The concept of bonding offers insight to Chinese seafarers in terms of their needs, perceptions and planning their seafaring careers in this sector.

Through multiple choice responses in relation to factors influencing their selection of foreign shipping company, Table 6 shows the range of common needs and priorities for seafarers. Good conditions of the vessel was ranked top by 85.5% of the respondents, followed by a comprehensive management system (78.9%) and safety culture (78.6%), whilst decent salary was ranked in 4th place (71.7%). It is worth noting that over two-thirds of the respondents expressed concern about the attention paid by the shipping company to their families, job security, food and welfare onboard, ranked from 5th to 7th respectively. These findings are confirmed by a representative of a Japanese ship company who highlighted the importance of a good welfare package in the Chinese context, with an emphasis on familial values, responsibility, and childhood education:

“If a ship company wants its crews to gain a feeling of belonging or loyalty to it, a number of conditions must be met. The first is the establishment of its enterprise culture. The second is taking care of seafarers’ families. The third is an annual pay system, family insurance, education fund for their children. So, the salary of your crews may not necessarily be the best in the labor market, but a comparative advantage in welfare package could still attract and retain commitment seafarers to work with you for long term.” (Capt L, 2017)

**Table 6:** Factors for selecting ship company by bonding

*No.*	*Item*	*No.*	*%*	*Bonding*	
				*No*	*Yes*
1	Vessel condition	272	85.5	*86.7*	*85.4*
2	**Management system**	251	78.9	**68.4**	**84.9**
3	**Safety culture**	250	78.6	**65.3**	**85.4**
4	Decent wage	228	71.7	*67.3*	*75.5*
5	**Care of families**	219	68.9	**62.2**	**73.6**
6	**Job security**	216	67.9	**58.2**	**73.1**
7	Food onboard	215	67.6	*68.4*	*67.9*
8	Promotion opportunity	192	60.4	*62.2*	*60.8*
9	Training opportunity	167	52.5	*51.0*	*53.8*
10	**Ship type/route**	162	50.9	**61.2**	**46.2**
11	Enterprise culture	111	34.9	*32.7*	*36.3*

*Notes*: The item “No” of bonding include “neutral” and “detached” in Table 5; bold indicates significance at *p* < .01

It is noted that career development factors including promotion and training opportunities were of concern to 60.4 and 52.5% of the respondents, respectively, and just over a half paid attention to the ship type and route. Interestingly, enterprise culture and communication factor were listed at the end, mentioned by a little more than one-third of the respondents (34.9%).

The boldened cells in Table 6 reflect significant statistical differences in responses, confirmed by T-Test analysis (*p* < .01). Distinctive needs among the respondents can be revealed from the different groups from the perspective of the bonding. Subsequently, a number of observations can be drawn:

First, differences between groups can be identified from different priorities in their needs. For instance, safety culture was listed as top priority by bonded seafarers, which is in contrast to their counterparts who favoured good conditions of the ship as the most important.

Second, differences between exported seafarers can be further recognised from other characteristics listed in Table 6. For instance, compared with others on vessel conditions and food/welfare onboard, bonded seafarers typically placed more emphasis upon the management system, seafarer families and job security and less to ship type and voyage route.

Moving to training opportunities, Table 7 shows that the vast majority (85.8%) of the respondents reported attending at least one of shore training courses. However, bonded seafarers reported more chances to attend training courses than others. With respect to differences between shipping companies in terms of investment on seafarer training, a small difference is noted in the number of the respondents who had yet to receive training (1.6% more respondents without Western shipping experience reported receiving no training compared to those with the experience). However, no significant impact of Western shipping experience is found on training opportunities, despite descriptive indications that those with Western shipping experience are more likely to encounter multiple training opportunities than those without.

**Table 7:** Training opportunities provided by the shipping company

*Category*	*Item*	*Not yet*	*Yes, once*	*Yes, many*
Bonding	Yes	14.5	17.4	68.1
	No	13.5	33.3	53.1
Western company?	Yes	13.1	13.1	73.8
	No	14.7	26.7	58.7
Total		14.2	23.0	62.8

With respect to which shore-based training courses are helpful, Table 8 shows that safety and technical training are ranked top (78%) and second (61%), respectively, followed by environmental protection, management systems, international regulation, and enterprise culture. While there was no significant difference between seafarers in terms of safety and technical training, bonded seafarers were likely to place more emphasis or appreciation on courses covering content such as environment protection, management systems, international regulations and enterprise culture.

**Table 8:** Use/helpfulness of training courses (%) by type of bonding (%)

***No.***	*Content*	*No.*	*%*	*Bonding*	
				*No*	*Yes*
1	Safety culture	248	78.0	79.6	77.8
2	Technic skills	194	61.0	56.1	64.2
3	Environmental protection	145	45.6	40.8	47.6
4	Management system	121	38.1	**28.6**	**42.9**
5	International regulations	108	34.0	**24.5**	**38.7**
6	Enterprise culture	66	20.8	**12.2**	**25.0**

Notes: bold as to indicate statistical significance

With respect to the impact of bonding on the retention and promotion of seafaring career, our survey shows that about three- quarters (73.7%) of the respondents confirmed a plan to end their seafaring career, of which 61% suggest a time frame of 5 years. A relatively small proportion of the respondents gave a clear indication regarding their position when consulted for the seafaring career, which accounts for less than 30%, leaving the majority of over 70% to ‘neutral’ or ‘depending’ responses. Further to differences of the respondents to above questions, Table 9 shows that those who indicated that bonded seafarers were significantly less likely than their counterparts in other groups to intend on ending their seafaring career, and giving negative advice on seafaring career to others.

**Table 9:** Planning to end of, or advising seafaring career by bonding (%)

*Item*	*End of seafaring career?*		*Advising seafaring career*		
	*Yes*	*No*	*Negative*	*Neutral*	*Positive*
Bonded	71.4	**28.6**	**11.9**	73.8	14.3
Neutral	77.6	**22.4**	**26.2**	67.2	6.6
Detached	88.2	**11.8**	**24.3**	64.9	10.8
Total	73.7	26.3	16.2	71.1	12.7

Given the importance of multicultural experience for the career development of Chinese seafarers, we asked the respondents to indicate their preferences in relation to multinational crewing patterns, with specific reference to all Chinese, mixed, or neutral. For a mixed or neutral choice, participants were asked which nationality of seafarers they preferred to work with. Generally, 43.3% selected “all Chinese”, 21.4% for “neutral”, 35.4% for “mixed”. Among those who select “neutral” or “mixed”, 66.7% gave their preference as Europeans, 47% to Filipinos, 16% to Indians, and 31% to others.

Regarding differences between groups in relation to multinational crewing patterns, Table 10 shows the variations by selected factors: age, education, seafaring length, rank, employment status, mobility experience, bonding, and others. It seems that the preference for multinational crewing patterns are more likely found among those who are younger (< 30 years old), bonded, university graduates, junior officers, crew agency seafarers, having past multinational working experience as well as those whose are currently working in Western or Chinese-background shipping companies. It is also clear that the preference for the all-Chinese crewing patterns was most related to those who were older (> = 40 years old), with greater seafaring experience (> 10 years), had a secondary education, were senior officers and ratings, were SOE employees and free seamen, were highly mobile and reported being no bonded, as well as those who didn’t already possess a multicultural working experience.

**Table 10:** Preference of multinational crewing pattern by selected factors

*Category*	*Item*	*No.*	*All Chinese*	*Neutral*	*Mixed*
Age band	< 30 years	84	25.0	33.3	**41.7**
	30–39 years	91	47.3	16.5	36.3
	> = 40 years	79	**58.2**	13.9	27.8
Education	University	56	28.6	21.4	**50.0**
	Vocational	121	40.5	19.8	39.7
	M. School	75	**57.3**	24.0	18.7
Seafaring length	<=5 years	86	32.6	25.6	**41.9**
	6–10 years	74	40.5	20.3	39.2
	> 10 years	92	**55.4**	17.4	27.4
Rank	Senior officer	86	**53.5**	15.1	31.4
	Junior officer	80	22.5	23.8	**53.8**
	Rating	88	**52.3**	25.0	22.7
Employment status	SOE	40	**52.5**	10.0	37.5
	Crewing agent	94	26.6	25.5	**47.9**
	Freeman	114	**55.3**	20.0	24.6
Mobility: crew agent	One only	122	32.0	23.0	**45.1**
	2–3 times	73	49.3	17.8	32.9
	> 3 times	21	**57.1**	19.0	23.8
Bonding	Yes	168	36.9	21.4	**41.7**
	No	81	**53.1**	22.2	24.7
Multinational crewing	Yes	148	31.8	19.6	**48.6**
	No	97	**57.7**	24.7	17.5
Ship manager	Wester	71	33.8	12.7	**53.5**
	Chinese	120	35.0	23.3	**41.7**
	Other Asian	77	46.8	23.4	29.9
Total		254	43.3	21.3	35.4

From the perspective of the shipping company, whether seafarers and cadets have multinational working opportunities appears largely dependent upon a trade-off between crew career development and manning cost. This is explained as follows:

“Comparing with the multinational crewing pattern for Chinese crews, many crewing agencies prefer a whole Chinese crewing pattern for two reasons: easier for management and more profits. For the purpose of seafaring career development, a multinational crewing pattern would be more beneficial to Chinese seafarers, but the shipping company may also give their preference to whole Chinese crews in order to reduce the manning costs.” (Capt. D, a Hong Kong ship owner representative)

With respect to the future of Chinese seafarer supply to international shipping, respondents were asked to offer their comments on the trend: growth, neutral or a decline. Generally, 37.8% shared positive views of growth, 31.5% negative views of decline, leaving 30.7% as neutral. As shown in Table 11, different groups held distinct views on the future of Chinese exporting seafarers. The positive view was more likely to come from those in the middle age bracket (30–39 years old), junior officers, and career-oriented, satisfied and attached seafarers, while negative prediction was more likely to be expressed by older (> = 40 years), senior officers and seafarers reporting an absence of bond.

**Table 11:** Views on the future of Chinese  exporting seafarers

Category	Item	No.	Decline	Neutral	Growth
Age band	< 30 years	74	21.6	37.8	40.5
	30–39 years	88	26.1	27.3	**46.6**
	> = 40 years	92	**44.6**	28.3	27.3
Rank	Senior officer	87	**43.7**	23.0	33.3
	Junior officer	72	26.4	31.9	**41.7**
	Rating	95	24.2	36.8	38.9
Bonding	Yes	177	25.4	31.6	**42.9**
	No	75	**45.3**	29.3	25.3
Total		254	31.5	30.7	37.8

## Research findings, conclusions and policy recommendations

This paper looked at the bond of Chinese-exported seafarers to international shipping companies and the subsequent impact on their career development. Based on interviews and survey findings, this report draws upon data analysis to identify features of bonding and the factors underpinning it, and the impact on their retention and career development in this sector. Accordingly, a number of research findings and conclusions are highlighted, as follows.

First, our survey indicates that the majority (58.7%) of the respondents did not report a bond with the shipping companies, but wished to develop such a feeling or close relationship with the latter. This can be shown as 68.4% reported feeling being a member, or desired to be a member, of foreign shipping companies. For Chinese seafarers, the bond is referred to as a relationship with shipping companies involving mutual trust, respect and long-term service. Nearly 20% (more precisely, 19.7%) were neutral in their responses (some of them even uncertain) and the remaining (11.9%) did not identify as sharing any bond.

Second, bonding cannot be narrowly understood as an economic matter. Instead, it reflects the need to recognise, consent or integrate into management systems, safety culture, and values of the shipping company workers are serving. It also reflects the seafarers’ desire for the shipping companies to respect and appreciate their contributions, and to promote career development and recognise the difficulties that their families face (especially with respect to family emergencies), so that they can provide longer term service. Different degrees of bonding were identified: 60% of the seafarers demonstrated “feeling like a member of the company”, while 40% expressed their desire to “have such a feeling”.

Third, it is confirmed that there is a relationship between bonding and the retention of Chinese crews: 77.2% of bonded respondents have been in long-term service with the same company; a value nearly 10% higher than the average of long-term servicing respondents. Furthermore, the respondents who signed onto long-term contracts with crewing agencies were 15% more likely to indicate a bond. Furthermore, we identified the following factors related to bond, including: education (vocational education in particular), multinational crewing experience, and cultural background of the shipping company (Chinese companies from Hong Kong, Singapore and Taiwan). However, highly mobile “free seamen” did not necessarily report weaker bonding. This finding is exemplified by the chief engineer in Section 3, who indicated having to leave to find a suitable shipping company to which he could share the same value or identity.

Fourth, the formation of bond and factors underpinning it can be further explored through the criteria in their preferences for selecting shipping companies. The survey results showed that 71.7% of the respondents were concerned about a “decent wage”, which was ranked at fourth behind the conditions of the ship, the management system and the safety culture of the individual shipping companies. More than two-thirds paid attention to whether the shipping companies looked at “taking care of crew families”, “job security” and “food and welfare onboard”, ranking fifth to seventh, respectively. The key difference between bonded and detached exported seafarers was the order or priority given to the factors above. For example, the former tended to prioritise safety culture while the latter did so with the “ship condition”. In addition, bonded seafarers appeared to pay less attention to “food welfare” than their detached counterparts.

Fifth, the formation and development of bonding was closely related to training investment of the shipping companies. For example, bonded respondents reported more opportunities to attend training courses, especially those related to management systems, international regulations and company culture.

Six, under the existing seafarer exporting system, the long-term cooperation between foreign shipping companies and Chinese crewing agencies was crucial in facilitating bonding which can be seen from two aspects: a) crewing agencies can recruit and manage Chinese crews on behalf of the shipping companies, a solid foundation for the formation and development of bonded seafarers. This can be illustrated by over-three quarters of “crewing agency seafarers” (i.e. signed long-term contract with crewing agencies) falling into bonded seafarers; b) the crewing agencies can serve as a channel for those highly skilled and bonded “free seamen” to access and service in partnership companies. The latter is mainly reflected in the fact that seafarers with high mobility were not necessarily people reporting weaker bonds, but indeed might be serving on a suitable shipping company for long-term service.

Seventh, bonding has an impact not only on personal seafaring careers but also on social contacts (e.g. relatives, friends, classmates, etc.). With regards to the former, bonded seafarers were less likely to plan the end of their seafaring career than their counterparts without bonds. Additionally, more than 40% of the bonded respondents preferred to work within a multinational crewing pattern, compared with nearly 60% of the respondents reporting no bond, who gave their preference to work within an all-Chinese crewing pattern. When consulted about following an exported seafarer career, bonded seafarers appeared to be cautiously supportive of the career path, contrary to those without bonds.

Eighth, with respect to the future of Chinese exported seafarers, nearly 40% of respondents were optimistic, with the bonded respondents expressing this optimism significantly more than detached counterparts.

Based upon the preceding research findings, we would like to offer the following policy recommendations to key stakeholders:

1. Foreign shipping companies: bonding is an important element of the company’s competitiveness so it is advisable to establish a partnership with Chinese crews by many measures, such as: increasing cadet opportunities for university graduates; career planning, multinational crewing patterns for cadets, shore-based job opportunities for excellent seafarers; taking care of their families, and allowing early disembarking in case of family emergencies; sharing company identity and values via various training courses; decent payment and bonuses; a special fund or loan programme in China for those outstanding Chinese young people.
2. Chinese crewing agencies: long-term cooperation with shipping companies prompting mutual trust and commitment to Chinese seafarers; strict implementation of contracts/agreements in terms of recruitment, training and boarding arrangement for Chinese crews; best service and support to Chinese crews to develop attachment and cope with the difficulties facing their families; jointly reject vicious competition at the expense of the decline of Chinese seafarers’ attachment.
3. Chinese exported seafarers: creating a favourable atmosphere for bonding among exported seafarers; making an effort to integrate into shipping companies; protecting/promoting the company brand by providing excellent service onboard; taking the initiative to communicate with shipping companies directly to reflect voices, opinions and requests of Chinese crews, and to ensure their legitimate rights, interests and need to be heard and respected.
4. Chinese government: bonding should become a part of the governmental strategy to develop seafarer supply and its “Belt and Road Initiative” by following measurements: encouraging training investment on Chinese seafarers; further opening the seafaring labour market to attract shipping companies; promoting good practices in the bonding between Chinese seafarers and shipping companies; encouraging foreign shipping companies to establish a special funding or loan programme in China to attract and provide training opportunities to committed younger people joining seafaring career.
5. International organisations (International Labour Organisation, International Maritime Organisation, International Chamber of Shipping, International Transport Workers’ Federation, etc.): the bond of seafarers to shipping companies should be listed as a theme of the International Seafarers’ Day in order to promote mutual trust, respect, and long term collaboration between shipping companies and seafarers, together with blacklisting of those shipping companies who treat seafarers poorly (e.g. delayed payment).

## Data Availability

The datasets used and analysed during the current study are available from the corresponding author on reasonable request.

## Footnotes

1 China Crew's Remuneration index [in Chinese] at: https://www.xindemarinenews.com/m/view.php?aid=27468, accessed on 15/04/2021.

2 The decline of the wage difference between shore and off-shore job is listed as one of most important factors contributing to the decline of the attractiveness of seafarer career in an internal document of the MoT recently: Framework for the research on the attractiveness of seafaring occupation (in Chinese).

## References

[CR1] BIMCO/ICS (2016). BIMCO-ICS manpower report: the global supply and demand for seafarers in 2015.

[CR2] Fan L, Fei J, Schriever U, Fan S (2018). An empirical study on the communicative competence of Chinese seafarers. Mar Policy.

[CR3] Ministry of Transport of China (MoT) (2020). China seafarer development report 2019 [in Chinese].

[CR4] Sampson H, Wu B (2007) Thoughts on safety: the views of Chinese seafarers, SIRC symposium proceedings, 138–156. Seafarers International Research Centre (SIRC)

[CR5] Tang L, Llangco MOS, Zhao Z (2016). Transformations and continuities of issues related to Chinese participation in the global seafarers’ labour market. Marit Policy Manag.

[CR6] Tang L, Zhang Z (2019). Global problems, local solutions: unfree labour relations and seafarer employment with crewing agencies in China. Ind Relat J.

[CR7] Wu B (2004). Participation in the global labour market: experience and responses of Chinese seafarers. Marit Policy Manag.

[CR8] Wu B (2006). Transformation from traditional to global seafarers: an assessment of Chinese seafarers in the global labour market, a keynote speech to the Shenzhen international forum for quality seafarers 19–20, 2006.

[CR9] Wu B, Lai KH, Cheng TCE (2006). Emergence of ‘new professionalism’ among Chinese seafarers: empirical evidence and policy implications. Marit Policy Manag.

[CR10] Wu B, Shen G, Li L (2007) The transformation of the Chinese labour market for seafarers. Seafarers International Research Centre (SIRC)

[CR11] Zhao Z, Walters D, Shan D (2020). Impediments to free movement of Chinese seafarers in the maritime labour market. Econ Labour Relat Rev.

